# Spatial modelling and inequalities of environmental noise in Accra, Ghana

**DOI:** 10.1016/j.envres.2022.113932

**Published:** 2022-11

**Authors:** Sierra N. Clark, Abosede S. Alli, Majid Ezzati, Michael Brauer, Mireille B. Toledano, James Nimo, Josephine Bedford Moses, Solomon Baah, Allison Hughes, Alicia Cavanaugh, Samuel Agyei-Mensah, George Owusu, Brian Robinson, Jill Baumgartner, James E. Bennett, Raphael E. Arku

**Affiliations:** a Department of Epidemiology and Biostatistics, School of Public Health, Imperial College London, London, UK; b MRC Centre for Environment and Health, School of Public Health, Imperial College London, London, UK; c Department of Environmental Health Sciences, School of Public Health and Health Sciences, University of Massachusetts, Amherst, USA; d Regional Institute for Population Studies, University of Ghana, Accra, Ghana; e Abdul Latif Jameel Institute for Disease and Emergency Analytics, Imperial College London, London, UK; f School of Population and Public Health, The University of British Columbia, Vancouver, Canada; g Mohn Centre for Children's Health and Wellbeing, School of Public Health, Imperial College London, London, UK; h Department of Physics, University of Ghana, Accra, Ghana; i Department of Geography, McGill University, Montreal, Canada; j Department of Geography and Resource Development, University of Ghana, Accra, Ghana; k Institute of Statistical, Social & Economic Research, University of Ghana, Accra, Ghana; l Institute for Health and Social Policy, McGill University, Montreal, Canada; m Department of Epidemiology, Biostatistics, and Occupational Health, McGill University, Montreal, Canada

**Keywords:** Environmental noise, Land use regression, Socioeconomic status, Road-traffic noise, Intermittency ratio, Sub-Saharan Africa

## Abstract

Noise pollution is a growing environmental health concern in rapidly urbanizing sub-Saharan African (SSA) cities. However, limited city-wide data constitutes a major barrier to investigating health impacts as well as implementing environmental policy in this growing population. As such, in this first of its kind study in West Africa, we measured, modelled and predicted environmental noise across the Greater Accra Metropolitan Area (GAMA) in Ghana, and evaluated inequalities in exposures by socioeconomic factors. Specifically, we measured environmental noise at 146 locations with weekly (n = 136 locations) and yearlong monitoring (n = 10 locations). We combined these data with geospatial and meteorological predictor variables to develop high-resolution land use regression (LUR) models to predict annual average noise levels (LAeq_24hr_, L_den_, L_day_, L_night_). The final LUR models were selected with a forward stepwise procedure and performance was evaluated with cross-validation. We spatially joined model predictions with national census data to estimate population levels of, and potential socioeconomic inequalities in, noise levels at the census enumeration-area level. Variables representing road-traffic and vegetation explained the most variation in noise levels at each site. Predicted day-evening-night (L_den_) noise levels were highest in the city-center (Accra Metropolis) (median: 64.0 dBA) and near major roads (median: 68.5 dBA). In the Accra Metropolis, almost the entire population lived in areas where predicted L_den_ and night-time noise (L_night_) surpassed World Health Organization guidelines for road-traffic noise (L_den_ <53; and L_night_ <45). The poorest areas in Accra also had significantly higher median L_den_ and L_night_ compared with the wealthiest ones, with a difference of ∼5 dBA. The models can support environmental epidemiological studies, burden of disease assessments, and policies and interventions that address underlying causes of noise exposure inequalities within Accra.

## Highlights

• Collected noise measurements in a SSA city in a large-scale campaign over 1-year.
• Used land use regression to model and predict environmental noise in Accra.
• Majority of the population exposed to noise levels above WHO guidelines.
• Poorest areas had the highest predicted noise levels on average.
• Models can support local environmental health assessments and policy making.

## 1 Introduction

Noise from anthropogenic activities is pervasive in urban settings and can have adverse effects on human health and wellbeing (European Environment Agency, 2020; Hammer et al., 2014; Kang, 2017a; World Health Organization, 2018). Epidemiolocal studies from cities in Europe and North America have shown that exposure to noise from road-, rail- and aircraft traffic sources can lead to a range health effects, including impacts on annoyance, sleep quality, cardiometabolic diseases, and impaired cognitive function (Basner and McGuire, 2018; Guski et al., 2017; Münzel et al., 2021; Thompson et al., 2022; van Kempen et al., 2018; Vienneau et al., 2019). Modelling and mapping the spread of environmental noise, mostly in high-income cities, has revealed highly unequal distributions across and within cities, sometimes patterned by socioeconomic gradients (Dale et al., 2015; Dreger et al., 2019; European Environment Agency, 2020). Within-city inequalities in noise exposure could also create and/or exacerbate existing health inequalities.

Cities in sub-Saharan Africa (SSA), home to some of the world's fastest-growing economies, are undergoing significant expansion and economic transformations. Growing SSA cities are now characterized by glaring urban transport problems, including traffic congestion, long commute times, and traffic related noise pollution (Amegah and Agyei-Mensah, 2017; Imoro Musah et al., 2020; Sietchiping et al., 2012). Traffic noise coexists with community/neighbor noise, such as loud, pervasive music from religious activities and informal/small businesses, making noise pollution in SSA cities an emerging health concern (Baloye and Palamuleni, 2015; Bediako-Akoto, 2018; Kazeem and Dahir, 2018; Wawa and Mulaku, 2015; Zakpala et al., 2014). Though common in European, North American, and increasingly in Asian cities (Aguilera et al., 2015; Council of the European Union, 2002; European Environment Agency, 2020; Liu et al., 2020; Walker et al., 2017; Wang et al., 2016; Xie et al., 2011) modelling and mapping of environmental noise to reveal levels and spatial variations are severely lacking within the SSA context. Thus, hindering local efforts to identify sources of noise, investigate health impacts, quantify burdens of disease, and design policies and interventions to mitigate noise and reduce inequalities in exposures. Furthermore, a major barrier for conducting a global burden of disease assessment due to environmental noise is a lack of exposure data in low- and middle-income countries, and thus generating estimates in these regions can contribute to that global effort.

Propagation models, which are based on mathematical description of emissions and transmissions of sound through the environment, have been widely used for modelling noise from road-, rail-, and aircraft-traffic sources, particularly in European cities (Garg and Maji, 2014; Khan et al., 2018). However, a challenge for implementing propagation models in many low and middle-income regions of the world has been that national governments or even international corporations do not routinely collect much of the data that are needed for the models, such as road-traffic counts, vehicle fleet compositions, or building height and footprint information with fine enough spatial or temporal granularity (Aguilera et al., 2015; Kang, 2017b; Sieber et al., 2017). Alternatively, land use regression (LUR) models (Hoek et al., 2008), which are commonly used for the estimation of spatial variability in air pollution within cities (Hoek et al., 2008), have also increasingly been applied to noise in recent years in some high and middle-income country cities (Aguilera et al., 2015; Alam et al., 2017; Chang et al., 2019; Drudge et al., 2018; Fallah-Shorshani et al., 2018; Harouvi et al., 2018; Liu et al., 2020; Raess et al., 2021; Ragettli et al., 2016; Walker et al., 2017; Wang et al., 2016; Xie et al., 2011). Currently, only one environmental noise LUR model has been developed in SSA, for informal settlements in South Africa (Sieber et al., 2017). A noise LUR model derives statistical relationships between measured noise metrics and predictor variables that represent a range of factors in the urban environment that are associated with the emission, propagation and attenuation of noise. Geospatial and meteorological predictor data (Hoek et al., 2008; Khan et al., 2018) are increasingly available globally; such as satellite derived land use measures (Larkin et al., 2017) or locations of road networks and human activities from OpenStreetMap (Barrington-Leigh and Millard-Ball, 2017). In urban SSA settings, where sources of noise are complex, LUR modelling is a cost-effective and attractive method for estimating noise, given that emissions (e.g., time-resolved traffic flows) and building canyon (e.g., building footprints and heights) data needed for propagation-based modelling are often not freely available or do not exist at all (Sieber et al., 2017).

To bridge the data and modelling gap of environmental noise in SSA cities and provide local data for policy formulation and environmental health assessments, we designed a LUR modelling study to predict and map spatial variations and inequalities in noise metrics within one of the largest and fastest growing metropolises in Africa. The models integrated noise data from a 1-year large-scale measurement campaign within the Greater Accra Metropolitan Area (GAMA) (Clark et al., 2020; Clark et al., 2021) with a suite of city-wide geospatial and meteorological data. The final models were used to predict long-term (annual) averages of noise levels representing different periods of the day (L_Aeq24hr_, L_den_, L_day_, L_night_) across the city. We also estimated census enumeration area population exposures and socioeconomic inequalities in noise levels within the Accra Metropolis (∼2 million people), the urban core of the GAMA. In a secondary analysis, we built regression models to explore whether the intermittency of the sound environment, represented by the intermittency ratio metric, was associated with the features of the environment and other noise metrics at measurement sites.

## 2 Material and methods

### 2.1 Study location

Our study was conducted in the GAMA (∼5 million people), the most densely populated area in Ghana. Accounting for over a fifth of the country's urban population, the region includes Accra Metropolis as its core (estimated population in 2010 and 2019: ∼1.66 million and ∼2 million) (Ghana Statistical Service, 2019) and the port city of Tema. The GAMA is the political, economic, and administrative capital of Ghana, and while these sectors drive urban economic growth, vast inequalities in income, housing and environmental quality remain (Annim et al., 2012; Dionisio et al., 2010; Fobil et al., 2010). As the population and city-limits have expanded over the years, demand for transportation has increased, with private vehicles or privately owned minibuses (known locally as trotro) as the main means of getting around (Imoro Musah et al., 2020). There is no train or tram services, and formal transit bus services are limited. Ride-shares such as Uber and Bolt, and motorcycle-taxis (‘Okada’), are more recently being used to complement the need for public transport (Acheampong et al., 2020). Noise pollution in particular, has been highlighted recently as an environmental health concern in local and international media (Bediako-Akoto, 2018; Kaledzi, 2018; Kazeem and Dahir, 2018; Knott and Gyamfi Asiedu, 2019).

### 2.2 Data

#### 2.2.1 Environmental noise measurement and metrics

**Fig. 1:**
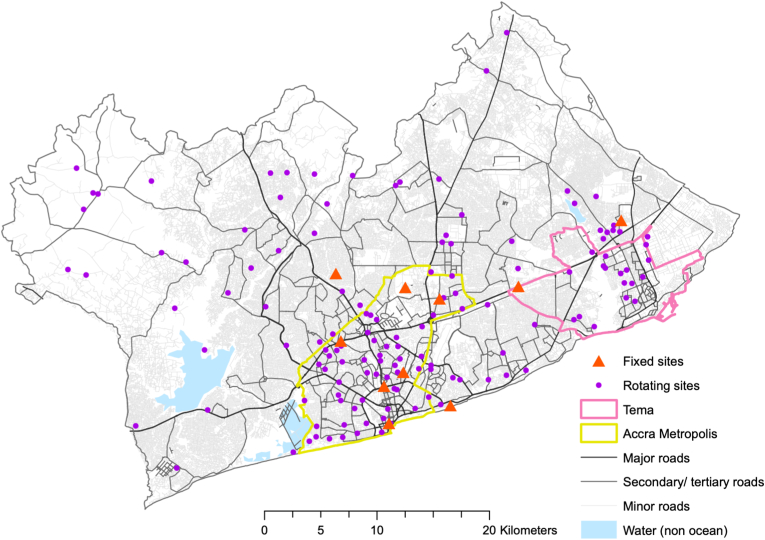
**Locations of rotating and fixed measurement sites in the Greater Accra Metropolitan Area (GAMA).** The GAMA, Accra Metropolis, and Tema boundaries are from the Ghana Statistical Service, road-network and water-body shapefiles are from OpenStreetMap (2019).

Between April 2019 and June 2020, we deployed sound level meters (SLM) near the roadside at 146 locations, comprising of 136 rotating (7-day) and 10 fixed (∼1-year) measurement sites (Fig. 1). The fixed sites represent diverse land use, socioeconomic, and transport features. Rotating sites were selected through stratified random sampling based on land use features and population data (World Bank, 2014). The measurement campaign was briefly interrupted in April–May 2020 during Accra's COVID-19 pandemic lockdown and subsequent COVID-related stoppages. The noise measurements have been described in detail in our previously published protocol paper (Clark et al., 2020).

We used Noise Sentry SLMs from Convergence Instruments (Québec, Canada) to continuously record A-weighted sound levels (decibels (dBA)) which were integrated and logged every minute. The Noise Sentry is rugged in design, built to withstand high temperatures, and the digital MEMs microphone is protected against water and dust, which is necessary for a setting like Accra. We deployed the SLMs in weather protective custom designed enclosures which we attached to poles or trees near the roadside at ∼ 4 m (±1 m) above ground, and at least 2 m away from the nearest façade. We undertook quality assurance and control (QA/QC) tests of SLM accuracy and precision throughout the campaign (Clark et al., 2020; Clark et al., 2021), which showed good agreement between the Noise Sentry SLMs and with a higher cost Type 1 SLM (Cirrus Optimus Red). Further details on the SLMs, data collection protocol, and QA/QC practices undertaken throughout the measurement campaign are described in the protocol paper (Clark et al., 2020). We calculated A-weighted equivalent continuous sound levels (L_Aeq,T_) for each site and date of measurement. Energy-based long-term average metrics, such as day-evening-night weighted (L_den_) and daytime (L_day_) and night-time (L_night_) noise levels are the mostly commonly used metrics in epidemiological studies and are robustly associated with a number of adverse health outcomes (Basner and McGuire, 2018; Clark et al., 2020; Guski et al., 2017; Thompson et al., 2022; van Kamp et al., 2020; van Kempen et al., 2018). As well, our previous descriptive study, which combined audio recordings with a deep learning acoustic classifier, found that road-transportation was a prominent sound source identified across measurement sites. Road-transportation sounds were particularly dominant in the city center (Accra Metropolis), and in commercial, business, and industrial areas (Clark et al., 2021). Therefore, throughout the paper and particularly with reference to Accra Metropolis, we refer to the measured and modelled data as environmental noise exposures, similar to previous noise LUR studies (Aguilera et al., 2015; Harouvi et al., 2018; Liu et al., 2020; Raess et al., 2021; Ragettli et al., 2016; Sieber et al., 2017; Wang et al., 2016; Xie et al., 2011).

#### 2.2.2 Predictor variables

We collected and collated spatial and temporal predictor variables that reflected factors in the urban environment associated with the emission, propagation, and attenuation of sound. Details of each predictor variable and its source are included in Table 1. To capture land use/land cover, we used a raster dataset at 20 m resolution that mapped four land cover classes across the GAMA from Spot 5 imagery attributed to the year 2014 (World Bank, 2014). To characterize vegetation, we calculated the Normalized Difference Vegetation Index (NDVI) from the spectral signatures of green vegetation from 30 m resolution satellite imagery. We obtained a Landsat 8 satellite product held on the U.S. Geological Survey department website attributed to a cloud free day (cloud cover: 0.02%) in January 2020. January was considered as a mid-point in the measurement campaign. Other days with Landsat 8 imagery were unusable for this purpose due to cloud cover over the area. However, there was minimal temporal variability of NDVI levels throughout the year due to Accra's location near the equator. To estimate building density, we made use of a high-resolution spatial dataset of building footprints attributed to the year 2019/2020 from Maxar/Ecopia (Price and Hallas, 2019), which we transformed into a dataset of building centroids (spatial center-point). This transformation was done due to the computational intensity of processing building footprints. To estimate human population density, we used population information from the most recent Ghana national census summarized within census enumeration areas (Ghana Statisical Service, 2010). Census enumeration areas are small geographic units with average population of 750–800 people and area 0.03–0.04 km^2^ within the GAMA. To capture road-traffic sources of noise, we used a road-network shapefile from OpenStreetMap (OSM) (OpenStreetMap, 2015) downloaded in 2019. OpenStreetMap is an open-source editable global database of urban geographic information which has grown rapidly over the years (OpenStreetMap, 2015). Barrington-Leigh et al. estimated that OpenStreetMap had 83% global coverage of roads as of 2016, and 45% coverage in Ghana (Barrington-Leigh and Millard-Ball, 2017). Though, we expect the road-network completeness in Accra in 2019 to be higher than the estimate for Ghana as Accra is a major capital city which would likely have higher coverage than other smaller cities/rural towns across the country, hence the lower country-wide average. As well, Barrington-Leigh et al. analysed data from 2016, and OSM is continually updated and improved overtime by users. To capture aircraft noise, we obtained the spatial boundaries of the Kotoka International Airport from Google Earth. For locations of human activity, we identified the latitude and longitude locations of churches, mosques, hospitals, primary and secondary schools, restaurants, shopping centers and markets, and bars/nightclubs from Google Places in 2019. We also obtained locations of bus stations/terminals from Google Places as an indicator of both human activity and road-transport sources. Finally, we retrieved data on elevation above sea level from a digital elevation model (DEM) for Africa at 90 m resolution (Verdin, 2017) and data on waterways from OSM (2019).

**Table 1:** Candidate predictor variables for LUR model selection.

Variable type	Categories	Spatial calculation	Source (Date)
Road-network (*Spatial line*)	Major roads; secondary/tertiary roads; minor roads; all roads	Total length within buffer (meters); Euclidean distance and square root distance to nearest (meters)	OpenStreetMap (2019) Barrington-Leigh and Millard-Ball (2017)
Airport (*Spatial polygon*) a	–	Euclidean distance and square root distance to nearest (meters)	Google Earth (2019)
Land cover (*raster*)	Industrial, business, commercial areas; informal high-density residential; formal residential; ‘other’ areas (e.g., forest, water, grassland, bare soil)	Area (meters^2^) within buffer	World Bank (2014) 20 m × 20 m
Locations of human activity (*Spatial point*)	Schools, hospitals, bus stations/terminals, restaurants, bars and nightclubs, churches, mosques, shopping centers	Presence/absence within buffer; count within buffer	Google Places (2019)
Normalized Difference Vegetation Index (*raster*)	–	Average value within buffer (range: 0–1; water (negative values) was omitted)	United States Geological Survey – Landsat 8 imagery −30 m × 30 m U.S Geological Survey (n.d.)
Human population density within enumeration areas (*Spatial polygon*)	–	Average value within buffer (pop/km^2^)	Ghana Statisical Service (2010)
Centroid of each building (*Spatial point*)	–	Count within buffer	Price and Hallas (2019)
Rivers/waterways (*Spatial line*)	–	Total length within buffer (meters)	OpenStreetMap (2019)
Elevation above sea level *(raster)* (meters)		–	U.S Geological Survey DEM (2017) (∼90 m) Verdin (2017)
Height of monitor off of the ground (meter)	–	–	Measurement campaign Clark et al. (2020)

Table includes all candidate predictor variables considered for the model selection process. The final models include a subset of these predictors which survived the model selection process.

a Aircraft traffic at Kotoka airport (∼1.8 million passengers a year) (Ghana Airports, 2017) is a fraction of what it is at other large airports in major cities such as Heathrow in London (∼80 million passengers/year) (Heathrow Airport, 2018) or Schiphol in Amsterdam (∼71 million passengers/year) (Heathrow Airport, 2018).

Variations in atmospheric conditions can affect acoustic wave propagation (i.e., atmospheric absorption) (Ghinet et al., 2019; Kang, 2017a; Truax, 1999), be sources of sound (e.g., rainfall), or influence human behaviors/activities that result in sound generation (Böcker et al., 2013). Thus, we collected time-resolved data on temperature (Celsius degree), wind speed (m/s), and relative humidity (%) at six of the fixed (∼yearlong) measurement sites throughout the campaign with small weather meters (Kestrel 5500, Nelsen-Kellerman, Pennsylvania, USA). We also retrieved daily rainfall (mm) data from the Ghana Meteorological Agency (GMA).

#### 2.2.3 Predictor data pre-processing

We created multiple buffers around the measurement sites which were based on the noise LUR literature (Aguilera et al., 2015; Ragettli et al., 2016): 50 m, 100 m, 200 m, and 500 m. We then mapped the spatial predictor variables to each buffer, centred by the coordinate location of the measurement site, through spatial overlay. We then clipped the spatial predictors so that only the features of the spatial predictors overlapping with each buffer remained. We calculated zonal statistics (e.g., average, sum, area) within each buffer, depending on the spatial predictor variable type (details in Table 1). Additionally, for distance variables we calculated the Euclidean distance from each monitoring site to the nearest major and secondary road and to the airport location and applied a square root transformation to capture potential non-linear relationships.

### 2.3 Model building and evaluation

We took a land use regression (LUR) approach to model and predict long-term average noise levels within the GAMA. Specifically, we constructed models for L_Aeq1hr_ and fit separate models for the day and night hours. In accordance with the Ghana Standards Authority, we defined the day-time as 6:00am–9:59pm (night-time: 10:00 pm - 5:59am) (Ghana Standards Authority, 2018). We assessed the linearity of the relationships between noise levels and the (continuous) predictor variables with bivariate scatter plots. We also initially built models which assumed (i) linear and (ii) non-linear associations (e.g., splines) between predictor and dependent variables and found that the predictive error between models was similar (Supplementary Information, Table S1). Therefore, we opted for the simpler modelling approach, which assumed linear relationships, and provided the added benefit of enhanced model interpretability. We additionally incorporated random intercepts for hour of the day to account for diurnal correlation of measured sound levels as well as random intercepts for site locations to account for any site-specific unmeasured variations.

Our model selection process was aimed at identifying parsimonious and generalizable models that also maximized predictive accuracy. We employed a two-step approach where we first chose the buffer radii for each predictor variable that had the highest correlation with the noise levels in each model. Consistent with other LUR modelling studies, we also considered the direction of the association with our *a priori* assumptions (Aguilera et al., 2015; Fallah-Shorshani et al., 2018; Lee et al., 2017; Ragettli et al., 2016; Sieber et al., 2017). Second, we used a stepwise forward model selection process to identify models with a reduced set of spatial predictor variables (Aguilera et al., 2015; Raess et al., 2021; Sieber et al., 2017). We began by inserting predictors which had the strongest bivariate associations with the noise levels (identified from the first step) and the process was stopped when the coefficient of determination (R^2^) was no longer improved by at least 1% (Aguilera et al., 2015; Chang et al., 2019). We considered removing predictor variables if their 95% confidence interval around the slope coefficients crossed zero; specifically, if the magnitude of the coefficient and the width of the confidence interval were large, we considered the estimate to be unstable and dropped the variable from the final model. The final models were then challenged by adding all excluded variables with their best buffer size (i.e., one buffer size per variable type) back into the models one by one to check if an improved model could be found. We also assessed whether there was collinearity present among predictor variables (r > 0.8), and if found, the predictor variable that was more correlated with the noise metric was retained in the model.

We evaluated the fit and external predictions of the final models with cross validation. We ran 10-fold cross-validation holding out data from 10% of random measurement sites (CV_10%sites_) and leave one site out cross validation (LOOCV). From the cross-validations, we calculated the median absolute errors, mean absolute errors, the mean errors, and the correlation of predicted and observed values (r and r^2^). Furthermore, we evaluated whether model assumptions were upheld using diagnostic plots to see whether the residuals were normally distributed and had random and constant variance. We also checked for any temporal and spatial patterns in the residuals. We checked spatial patterns by visualizing the residuals in variogram plots and calculated the Moran's I statistic of spatial autocorrelation. Finally, we evaluated potential multicollinearity in the final models as a whole using variance inflation factors (VIF).

#### 2.3.1 Sensitivity analyses

Since the primary aim of this analysis was to predict annual average noise levels across the GAMA, we could not use the weather data for spatial prediction as we only collected it at six sites in the city. However, we conducted sensitivity analyses to estimate the associations of noise levels with time-resolved weather conditions, specifically wind speed, temperature, relative humidity, and rainfall, and to investigate whether their inclusion in the main spatial models improved predictive accuracy. We additionally modelled the final predictor variable sets with Random Forest models as a sensitivity analysis for the choice of model infrastructure. Random Forest models have been shown previously to improve predictive accuracy over linear regression in a noise LUR study conducted in Canadian cities (Liu et al., 2020).

### 2.4 Predicted noise level surfaces

We made predictions of annual average noise levels for each hour of the day for an ~50 m × 50 m surface of unmeasured locations in the GAMA. Predictions of LAeq_1hr_ were made onto 24 surfaces, each representing an hour of the day. From the 24, hourly surfaces, the LAeq_24hr_ (the 24-h equivalent continuous sound level), L_den_ (day-evening-night sound level, a descriptor which penalizes 10 dBA for night-time and 5 dBA for evening noise), L_day_ (day-time sound level), and L_night_ (night-time sound level) metrics were logarithmically calculated. L_day_ was calculated with respect to day-time hours between 6:00–21:59 and L_night_ between 22:00–5:59. L_den_ was calculated with respect to day-time hours between 6:00–18:59, evening between 19:00–21:59, and night-time between 22:00–5:59. We restricted predictions to areas which represented the measurement sites so that we did not predict out of sample. Thus, we excluded areas that were covered by waterbodies, and/or areas that were fully grassland/forest (i.e., did not contain any roads).

### 2.5 Population exposure to noise levels in Accra Metropolis

We estimated the percentage of the population exposed to different levels of noise in Accra Metropolis. We first spatially overlayed the predicted noise level surfaces onto a map of enumeration areas from the 2010 census in Accra Metropolis (Fig. S1). Enumeration areas reflect the location of residence at the time of the census and the smallest spatial administrative unit in Ghana. We then calculated average noise levels within each enumeration area and estimated the number of people exposed to varying noise levels of 5 dBA increments (e.g., 50–54 dBA, 55–59 dBA, etc.) based on the population distribution in 2010.

### 2.6 Socioeconomic inequalities of enumeration area noise levels in Accra Metropolis

We investigated whether noise levels in Accra Metropolis were associated with measures of enumeration area socioeconomic status (SES). Measures of consumption levels ($) (Deaton, 1992; Deaton and Zaidi, 2002) and inequalities in consumption may be a cause of, and/or a result of, physical and social environments, opportunities, access, security, and empowerment within households and communities. Importantly, consumption is a reflection of what people can afford and can access. Thus, our main measure of area-level SES was median log equivalized household consumption (Ghanaian Cedi (GH₵)) within each enumeration area. Household consumption was first derived from total annual real household expenditures and rent captured within the 2012–2013 Ghana Living Standards Survey (GLSS) (93% response rate) (Expenditures: food, beverages and alcohol, tobacco, clothing and footwear, housing, electricity, water, gas and other fuels, furnishings, equipment, routine maintenance, health, transport, communications, recreation and culture, education, hotels, cafes and restaurants, and miscellaneous goods and services). To then estimate median household consumption within all enumeration areas, we combined the GLSS with the 100% sample of the most recent census (2010) in small area estimation models (Corral et al., 2020; Elbers et al., 2003) which derived relationships between consumption, area and other factors such as asset ownership, education, employment, housing quality, and socio-demographics. It is worth noting that the datasets used to predict SES (i.e., consumption) and noise levels are independent. As secondary SES measures, we used census data on the number of individuals within each enumeration area with post-secondary education (education measure) and the number of unemployed individuals (unemployment measure). These measures are aggregates of household level SES, individual education and unemployment, summarized at the enumeration area-level, and represent proxies for area-level SES measures.

We estimated Pearson correlations between enumeration area SES measures with noise levels and summarized noise distributions across quintiles of the SES measures (5 groups, 20% of enumeration areas in each group). We further investigated whether differences between groups were statistically significant using difference-in-means tests with a p-value cut-off of <0.05.

### 2.7 Predictors of urban sound intermittency

There is emerging evidence that the degree of noise intermittency, characterized by the intermittency ratio metric, can be independently associated with some adverse health outcomes, or can act as an effect modifier of the relationship between noise levels and health outcomes (Brink et al., 2019b, Brink et al., 2019a; Eze et al., 2017; Foraster et al., 2017; Thiesse et al., 2020; Vienneau et al., 2022). Therefore, we conducted a secondary analysis and examined intermittency ratio metrics at each measurement site. The intermittency ratio is defined as the percentage of sound energy in the total energetic dose that is created from distinct sound events that exceed a threshold (Wunderli et al., 2016). Following the calculation procedure in Wunderli et al. (2016) (Equation in S1), we used a threshold of +3 dBA above the L_Aeq,T_ for the time period to calculate intermittency ratios for the day (IR_day_; 6:00am–9:59pm) and night-time (IR_night_; 10:00pm–5:59am) periods for each site. We then followed the same model building process as described in Section 2.3 to identify the potential environmental factors associated with the degree of day and night-time intermittency ratios at the measurement sites. For these models, day and night-time noise levels (L_day_, and L_night_) were additionally incorporated into the modelling process as predictors, and we post-hoc evaluated whether land use classifications assigned to each measurement site by the field team (see (Clark et al., 2021) for details) modified the associations of the predictor variables in the final models through interaction terms.

Analyses were conducted in R (R version 3.6.3) and some data visualizations using ArcMap ® software by Esri (Version 10.8).

## 3 Results

### 3.1 Noise level LUR model performance and predictor variable associations

The final models included between five and six spatial variables (Table 2). LAeq_1hr_ was positively associated with road-traffic predictors (i.e., length of major roads, length of secondary/tertiary roads) and the presence of restaurants, and negatively associated with variables representing vegetation (NDVI) and formal low/medium residential land use. The variables which explained the most semi-partial variance in the fixed effect component of the LAeq_1hr_ models were NDVI and length of major roads.

**Table 2:** Mean associations of noise levels with spatial predictor variables in the final LUR models^a^.

Model	Predictor variables	Predictor variable unit	Buffer size (m)	Coefficient **[95% confidence interval]**
LAeq_1hr_ for all day-time hours (dBA)				
	*Intercept*	–	–	65.2 [64.2, 66.3]
	Total length of major roads	Standardized^b^ (meters)	100	2.5 [1.2, 3.7]
	Total length of secondary/tertiary roads	Standardized (meters)	200	1.8 [0.9, 2.8]
	Formal low/medium residential area	Standardized (meters^2^)	200	−0.8 [-1.5, −0.1]
	Normalized difference vegetation index	Standardized (value)	50	−2.8 [-3.7, −2.0]
	Number of restaurants	Count	100	1.4 [0.5, 2.4]
	Population density	Standardized (people/km^2^)	500	0.8 [-0.4, 2.0]
LAeq_1hr_ for all night-time hours (dBA)				
	*Intercept*	–	–	57.2 [55.2, 59.2]
	Total length of major roads	Standardized (meters)	100	3.0 [1.7, 4.4]
	Total length of secondary/tertiary roads	Standardized (meters)	200	2.2 [1.2, 3.3]
	Formal low/medium residential area	Standardized (meters^2^)	200	−1.3 [-1.9, −0.5]
	Normalized difference vegetation index	Standardized (value)	50	−2.2 [-2.9, −1.5]
	Number of restaurants	Count	100	1.8 [0.8, 2.9]

a Models incorporated random effects for *site* and *hour of the day*. Mean associations of spatial predictor variables were adjusted for monitor height in the model. The coefficients of predictor variables in the main models had the same direction in bivariate models (Table S4).

b Continuous variables were standardized by subtracting the data mean and dividing by the data standard deviation. A 1-point change in a standardized variable corresponds to a 1 standard deviation increase on the original scale.

The median absolute errors (MAEs) of the final LAeq_1hr_ models ranged from 2.9 to 3.4 dBA with CV_10%sites_ and the correlation of predicted and observed values (r) ranged from 0.72 to 0.74 (r^2^: 0.51 to 0.54). The mean error (ME), a measure of bias, was close to zero, indicating no systematic under or over prediction (Table 3). Results from LOOCV were very similar. We did not find evidence that model assumptions were violated, and model residuals were randomly distributed. Furthermore, Moran's I for the residuals indicated a tendency towards spatial randomness (Range of model's Moran's I values: −0.06 to 0.05). Variance Inflation Factors in the final models were low, between 1.0 and 2.0, indicating very low or no correlation among the variables in the final models that could inflate the coefficients. As a sensitivity analysis, we used Random Forest models to generate predictions using the final predictor variable sets and found no improvement in MAE (Table S2). As an additional sensitivity analysis, we included time-resolved weather variables into the final spatial models and found significant associations between weather variables and LAeq_1hr_, but no improvement in the overall model predictive accuracy (Table S3).

**Table 3:** Final noise level LUR model prediction accuracy from 10-fold 10% random site hold-out cross validation (CV_10%sites_).

Model	r	r^2^	Median absolute error	Mean absolute error	Mean error
LAeq_1hr_ for all day-time hours (dBA)	0.74	0.54	2.92 dBA	3.60 dBA	−0.34 dBA
LAeq_1hr_ for all night-time hours (dBA)	0.72	0.51	3.38 dBA	4.01 dBA	−0.41 dBA

r^2^ approximates R^2^.

### 3.2 Spatial patterns of noise levels in the greater Accra Metropolitan area

**Fig. 2:**
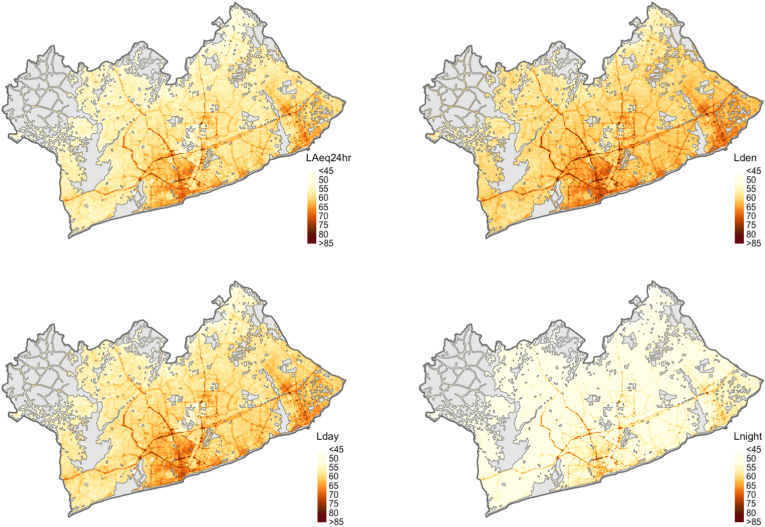
**Predicted noise levels in the Greater Accra Metropolitan Area.** Predictions were made for a fixed height of 4 m off the ground onto an ~50 m × 50 m grid of the city and calculated from the 24 surfaces of long-term hourly averages. Grey areas on the map represent areas excluded from prediction as they are out of sample (e.g., water bodies, forest/grassland). Legend: LAeq_24hr_ (dBA): 24-h equivalent continuous A-weighted noise level; L_den_ (dBA): Day-evening-night equivalent continuous A-weighted noise level. L_den_ was calculated with respect to day-time: 6am-6pm (13 h); evening: 7pm–9pm (3 h); night-time: 10pm-5am (8 h); L_day_ (dBA): Day-time equivalent continuous A-weighted noise level (6am-9:59 pm); L_night_ (dBA): Night-time equivalent continuous A-weighted noise level (10:00pm-5:59 am).

Spatial patterns of day and night-time noise levels in the GAMA were nearly the same, though day-time noise levels were higher by approximately 7–8 dBA (Fig. S2). Accra Metropolis, the most populated and urbanized area of the GAMA, had some of the highest predicted L_den_ (median: 64 dBA), as well as the port city of Tema in the east of GAMA (median: 62 dBA) (Fig. 2). Predicted L_den_ was highest near major roads (median: 69 dBA), followed by secondary/tertiary roads (median: 63 dBA), and then near minor roads (median: 60 dBA) (Table 4). The peri-urban periphery in the north and west of the GAMA had the lowest levels of L_den_ (median: 58 dBA) and L_night_ (median: 50 dBA) (Fig. 2).

**Table 4:** Predicted noise levels in the Greater Accra Metropolitan Area (GAMA), Accra Metropolis, and stratified by road-networks.

	LAeq_24hr_ (dBA)	L_den_ (dBA)	L_day_ (dBA)	L_night_ (dBA)
**GAMA**	57.0 (54.8, 59.3)	60.2 (58.2, 62.4)	58.5 (56.1, 60.7)	51.2 (49.6, 53.3)
**Roads**^a^				
Major roads	65.1 (61.8, 68.4)	68.5 (65.2, 71.8)	66.4 (63.0, 69.8)	59.9 (56.6, 63.4)
Secondary/tertiary roads	60.3 (57.5, 63.3)	63.4 (60.9, 66.4)	61.7 (58.8, 64.7)	54.3 (52.3, 57.2)
Minor roads	57.2 (55.1, 59.4)	60.3 (58.4, 62.4)	58.5 (56.3, 60.8)	51.3 (49.7, 53.2)
**Accra Metropolis**	61.2 (58.0, 64.2)	64.1 (61.1, 67.0)	62.7 (59.4, 65.6)	54.4 (51.8, 57.4)

Data summarized as median and interquartile ranges (IQR).

a 100 m buffers were created around each road type and average noise levels were calculated amongst all the points within the 100 m buffers corresponding to each road-type.

### 3.3 Population exposures to noise in Accra Metropolis

**Fig. 3:**
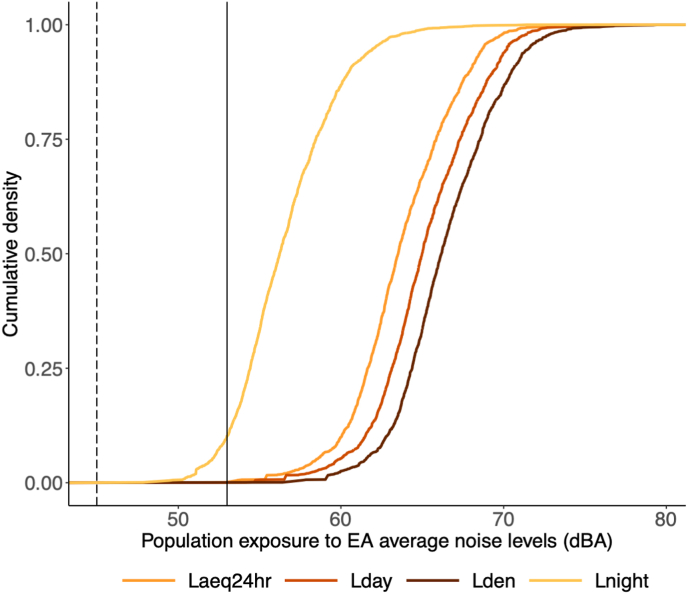
**Cumulative densities of the proportion of the Accra Metropolis population living in enumeration areas (EA) with varying noise levels.** The solid grey vertical line and the dashed black vertical line shows the L_den_ and L_night_ limits for road-traffic noise based on WHO guidelines for the European region, respectively (World Health Organization, 2018).

Almost the entire population in the Accra Metropolis lived in enumeration areas where the average L_den_ and L_night_ exceeded the WHOs (European) guidelines for road-traffic noise (L_den_: 53 dBA; L_night__:_ 45 dBA) (World Health Organization, 2018) (Fig. 3) and furthermore exceeded 55 dBA L_den_ and 50 dBA L_night_. The majority of the population in the Accra Metropolis lived in enumeration areas with average L_den_ of 60 to 64 dBA (31%, 515,873 people) or 65 to 69 dBA (53%, 876,098 people) and average L_night_ of 55 to 59 dBA (54%, 888,181 people) (Table S5, Fig. S3). With a recent projection of around 2 million people in Accra Metropolis in 2019, we expect the current numbers of people exposed to be higher than our estimates which are based on the 2010 census.

### 3.4 Area-level socioeconomic inequalities of noise in Accra Metropolis

**Fig. 4:**
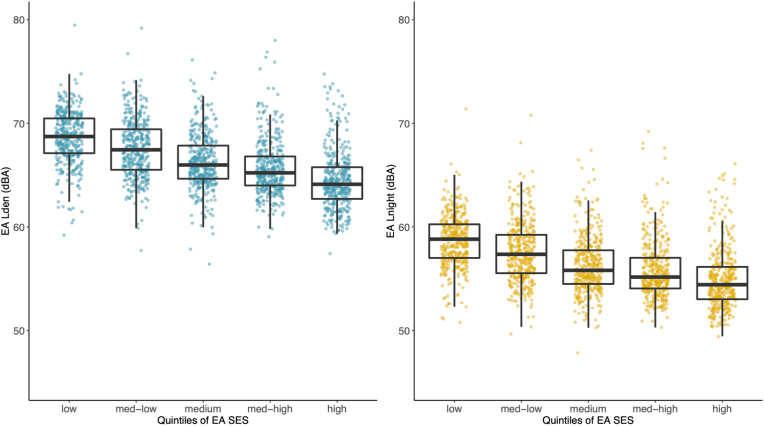
**Distribution of enumeration area (EA) L**_**den**_**and L**_**night**_**across quintiles (20% increments) of EA socioeconomic status (SES) in Accra Metropolis.** SES: EA median log equivalized household consumption. The upper and lower limits of the black box represent the interquartile range of the distribution and the horizontal line within the box represents the median. Each colored point represents an EA average noise level (dBA).

We observed an inverse relationship between enumeration area noise levels and our primary metric of SES (consumption) in Accra Metropolis. The poorest enumeration areas (bottom 20% of SES distribution) had statistically significant (p < 0.01) higher L_den_ (median: 69 dBA) compared with the wealthiest enumeration areas in the top 20% (median: 64 dBA) with a stepwise gradient for enumeration areas in between (Fig. 4). The same trend held for night-time noise levels. Though, even within a SES quintile, there was considerable variation in noise levels.

An inverse, but slightly weaker, relationship was found for noise levels and the number of individuals with post-secondary education within enumeration areas (Table 5). The enumeration areas in the lowest quintile of this distribution had a median L_den_ of 69 dBA compared with the wealthiest enumeration areas at 65 dBA. The weakest relationship was found for the number of unemployed individuals in enumeration areas (Table 5).

**Table 5:** **Correlation between enumeration area (EA) L**_**den**_**and L**_**night**_**and EA socioeconomic status metrics.** Table shows Pearson correlation coefficients and 95% confidence intervals around correlation estimates.

	Median log equivalized household consumption	Number of individuals with post-secondary education^a^	Number of unemployed individuals^a^
**L**_**den**_	−0.45 [-0.49, −0.41]	−0.34 [-0.38, −0.30]	−0.21 [-0.25, −0.17]
**L**_**night**_	−0.39 [-0.43, −0.36]	−0.29 [-0.33, −0.25]	−0.18 [-0.23, −0.14]

a The relationship was the same between the *number* and the *percentag*e of individuals with post-secondary education or who were unemployed within each enumeration area.

### 3.5 Predictors of intermittency ratios

Intermittency ratios for both the day and night-time hours were negatively associated with predictor variables representing roads with large and constant traffic flows, such as the length of major and secondary/tertiary roads within buffers around measurement sites (Table S6). However, the intermittency ratio for the day-time hours was positively associated with the length of minor roads within buffers, likely capturing sparse and intermittent sounds of road-traffic on these types of roads (Table S6). NDVI was positively associated with the intermittency ratio in the day-time hours, possibly due to the low background sound levels in areas with higher vegetation, and thus the ability of day-time intermittent sound events to emerge from background. In both the day and night-time models, noise levels (L_day_ and L_night_) were significantly positively associated with intermittency ratios, and the magnitudes of the associations were modified by land use classifications at each measurement site (Table S6).

## 4 Discussion

Environmental noise has been increasingly recognized as an environmental exposure of public health importancein growing SSA cities. However, there is scarce city-level data on environmental noise exposure to aid local policy and decision making or investigate and quantify health effects. Our study is the first of its kind in SSA to model, map, and investigate city-wide socioeconomic inequalities of predicted environmental noise exposures within a major African metropolis. We found that nearly all areas in the GAMA had L_den_ and L_night_ levels which exceeded international guidelines. The highest levels were in the city center and near major roads. Noise levels were not equally spread across neighborhoods as we found evidence that lower SES neighborhoods generally had higher levels compared with their wealthier counterparts.

Noise levels in Accra were positively associated with traffic-related variables, particularly major roads (highways, motorways), similar to previous LUR studies in North America (Fallah-Shorshani et al., 2018; Liu et al., 2020; Ragettli et al., 2016; Walker et al., 2017), Europe (Aguilera et al., 2015; Alam et al., 2017), Asia and the Middle East (Chang et al., 2019; Harouvi et al., 2018; Wang et al., 2016), and South Africa (Sieber et al., 2017). Multi-lane and higher-speed roads can facilitate higher traffic volumes, and attract a fleet composition with a higher percentage of heavy vehicles that can produce higher noise levels in these areas (Curran et al., 2013). The mechanisms by which motor vehicles generate noise is multi-faceted (Kang, 2017a) and include engine sounds, tire contact with the road and driver behavior such as honking (Vijay et al., 2015). Thus, interventions to reduce road-traffic noise can take on many forms, including vehicle emissions reduction (e.g., modifications to engines and tire materials), land use planning and transport management (e.g., separation between roads and buildings), the modification or creation of structures such as noise barriers or green vegetation (Curran et al., 2013; Kang, 2017a), and behavioral change interventions (e.g., ban on horns/honking) (Ali and Tamura, 2003; Gettleman, 2020; Vijay et al., 2015). In Accra, it was estimated that 20% of roads are still unpaved, particularly in the poorer neighborhoods (ASIRT, 2014); thus modifying pavement material (Curran et al., 2013; Donavan, 2005) could potentially reduce some road-transport noise, particularly on higher-speed roads (Eurocities, 2015). As well, given the dominance of transport by private vehicles, the local government in Accra could consider changes to urban design, placement of key services, safety measures and public messaging that inspire modal shifts towards cycling and walking and mass transit, as a mechanism to reduce road-traffic noise in the city. These interventions also have added benefits related to reductions in vehicular air pollution and greenhouse gas emissions and an increase in physical activity through active transport (Giles-Corti et al., 2010; Nieuwenhuijsen, 2021). Recent measures to curb vehicular air pollution emissions in Ghana, such as the regulation and taxes imposed on the import of old (and often noisy) vehicles into the country, may have an indirect impact on road-transport related noise.

Noise levels were generally higher in the city-core (Accra Metropolis) and in industrial (Tema Metropolis) areas (Drudge et al., 2018; Harouvi et al., 2018; Ragettli et al., 2016; Sieber et al., 2017) compared with outlying peri-urban and formal residential areas, where vegetation, which is a natural noise attenuator (Halim et al., 2015), was more abundant. Previous research on SSA cities suggests that outdoor noise sources in these settings extend beyond road, rail, and aircraft transportation and can include outdoor religious activities, social events/gatherings, formal and informal commercial activities, and large and small-scale industrial activities (Olayinka, 2012; Samagwa et al., 2010; Zakpala et al., 2014). Additionally, the number of restaurants in an area, which may serve as a proxy for general ‘neighborhood’ sources of noise and commercial activities in our models, was positively associated with noise levels. Previous research from South Africa found that L_den_ levels were significantly positively associated with high neighborhood noise annoyance (Sieber et al., 2018). It is also common for restaurants in residential and commercial areas of Accra to play music from loudspeakers, and many restaurants in the city are ‘open-air’ concept, providing nearby residents with little protection from exposure to sound generated by the restaurants. Previous research from Tanzania studied noise at restaurants and found elevated levels both indoors and outdoors (Samagwa et al., 2010), in part due to music being played. The perception of different types of city sounds can vary widely between countries and are intricately linked to social, cultural, and contextual factors related to the time and place in which the sound is perceived, as well as personal preferences and demographics (Deng et al., 2020; Kang, 2010, 2017c). Beyond a few small studies in SSA related to religious noise making (often accompanied by loud music) (Armah et al., 2010; Zakpala et al., 2014), and music from commercial shops (Ebare et al., 2011), there is scarce research exploring whether elevated human speech and outdoor music sounds, generated within a restaurant environment, would be considered unwanted noise or just ‘sounds of city life’ by the local population in Accra. Therefore, future soundscape research studies conducted within this understudied environment would shed light on local perceptions of different types of sounds, and situations and circumstances which impact perception.

Almost all areas within 100 m of major and secondary/tertiary roads and within the Accra Metropolis (main city center), where road-traffic noise sources are highly prevalent (Clark et al., 2021), had predicted noise levels which exceeded the World Health Organization (WHO) guidelines for road traffic noise (L_den_ (53 dB), L_night_ (45 dB)) (World Health Organization, 2018). Chronic exposure to road-traffic noise beyond these guideline thresholds is associated with adverse health effects including sleep disturbance, annoyance, and cardiovascular diseases. While the guidelines were developed for the European region, with the majority of the evidence underpinning them from European and North American countries, the WHO report does state that the guidelines can be considered applicable in other regions and suitable for a global audience (World Health Organization, 2018). Furthermore, we found that almost the entire population in Accra Metropolis (∼2 million people in 2019) lived in areas where L_den_ and L_night_ exceeded 55 and 50 dBA, respectively. Based on evidence from WHO commissioned systematic reviews (2018) (Basner and McGuire, 2018; Guski et al., 2017), at and above these noise levels over 11% (11–49%) and over 4% (4–12%) of the population are likely to be highly annoyed (L_den_) and sleep disturbed (L_night_) from road-traffic noise exposure, respectively (World Health Organization, 2018). As noise epidemiological research is currently lacking in Africa, future research in Accra can utilize our noise exposure surfaces to generate local evidence of the health effects of noise; this will have the effect of strengthening and diversifying the global literature base on noise health effects around the world.

We found that in Accra, the poorest enumeration areas had higher median L_den_ and L_night_ levels compared with the wealthier ones. Previous noise studies conducted in Europe, North America, and China using SES measures derived from material deprivation indicators, such as income, deprived living area, or mean dwelling value found similar trends (Casey et al., 2017; Dale et al., 2015; European Environment Agency, 2020; Lam and Chan, 2008). For our education measure, an indicator which may also reflect behavioral aspects, median noise levels were similarly lower in enumeration areas with a higher number of individuals with post-secondary education. Our analysis of area-level education and noise reflects results from a similar study in Montreal Canada (Dale et al., 2015). Though this relationship has had mixed results among studies that looked at individual-level associations in Europe (Dreger et al., 2019). In Accra, poorer communities are likely burdened by multiple environmental pollutants in addition to noise, as previous studies have found higher levels of PM_2.5_ air pollution concentrations in lower SES neighborhoods (Dionisio et al., 2010; Zhou et al., 2011).

In a secondary analysis, we calculated intermittency ratios for each measurement site and explored the potential associations of environmental features and noise levels within LUR models. The challenge of modelling a metric, such as the intermittency ratio, with spatial LUR models is that the predictor variables are often temporally static, thus making it difficult to capture intermittency which is inherently time dependent. Predictors which vary in both time and space may have allowed us to capture noise intermittency better. Furthermore, the medium-low predictive accuracy of the intermittency ratio models could be due to the measurement of the metric itself. We calculated intermittency ratios with sound level data integrated every minute; thus, we would have missed some infrequent sound events/peaks that would have been lost in the integration. We can also only interpret our results within the context of the fixed event cutoff that we used (+3 dBA). If we had modelled intermittency ratios with stricter cutoffs, the estimated intermittency ratios in the GAMA would be lower (Clark et al., 2021).

### 4.1 Strengths and limitations

Our research is one of the first to develop a LUR model of noise in SSA (Sieber et al., 2017) and the first to do so in West Africa. The models incorporated a suite of geospatial predictor variables and leveraged noise measurements from a large-scale and long-term data collection campaign. Finally, the comparison of predicted noise levels with small area SES measures is the first study to our knowledge to characterize inequalities of noise in a SSA setting. These models and the predicted noise surfaces provide opportunities for major environmental epidemiologic studies that would provide locally sound and globally relevant data on noise health effects within this understudied region. The noise exposure surfaces can also be used to conduct environmental burden of disease assessments, which can feed into local noise policy and decision making.

Our research has several limitations. While we did include a wide variety of spatial predictor variables in the study, we were not able to obtain spatially and/or temporally resolved information on traffic volume and fleet composition. Inclusion of this information could have improved model predictive accuracy, particularly from traffic-related sources. Further, we were not able to capture potential small-scale variations in noise propagation due to sound reflection or absorption in the built environment as we did not have data for building height and material and ground material (Kang, 2017a). We also made the assumption that the spatial predictors were stationary in time and representative of the period when the noise measurements were taken. This assumption may not be true for all spatial predictors as the census data which was used to estimate population counts was generated in 2010 and the dataset used to estimate land cover dates to 2014. The temporal misalignment of some of the predictor variables may be especially relevant for a rapidly urbanizing context such as the GAMA, and particularly its peripheries outside of the city center. With respect to SES inequalities of noise, our analysis was at the enumeration area level, and we recognize that associations at the individual level may be different. There is also a temporal misalignment between the noise and SES data. We used SES metrics estimated from the 2012 GLSS and the 2010 census as they were the most recent data of its kind, though the noise data were collected in 2019/2020. It is possible that the spatial distribution of SES in some parts of the GAMA in 2010 differ to present day realities (2019/2020). Though we expect this to be minimal in the city center (Accra Metropolis) where we conducted the SES analysis, as the major changes to within city migration, land use, and urban planning, are taking place at the peripheries of the GAMA (Addae and Oppelt, 2019). Future work incorporating the 2020 census is warranted to verify if trends have remained the same or changed. The 2020 census was delayed due to the COVID-19 Pandemic but may be completed and data released in a few of years.

## 5 Conclusion

The measured and predicted noise levels exceeded international health-based guidelines almost everywhere in the Greater Accra Metropolitan Area. At these levels, it is likely that common adverse health impacts attributable to environmental noise exposures, such as annoyance, sleep disturbance, and cardiovascular diseases, are experienced within the city. Furthermore, noise levels varied unequally across the city and poorer neighborhoods were generally worse off in terms of noise levels than the wealthiest neighborhoods. The spatial and social inequalities in environmental noise in Accra further highlight the need for local government to consider the equity impacts of urban planning and policy decision making. This is particularly the case as inequalities in noise exposure, compounded with socioeconomic inequalities and other environments exposures (e.g., air pollution), could further entrench health inequalities in Accra. City-level actions are needed to tackle this environmental exposure in Accra though changes in infrastructure, services and regulations that could also have broader and equitable benefits for health and wellbeing.

## Credit Author Statement

Sierra N Clark: Conceptualization, Data curation, Formal analysis, Funding acquisition, Methodology, Project administration, Visualization, Writing – original draft, Writing – review & editing. Abosede S Alli: Data curation, Project administration, Writing – review & editing. Majid Ezzati: Conceptualization, Methodology, Supervision, Funding acquisition, Resources, Writing – review & editing. Michael Brauer: Methodology, Supervision, Writing – review & editing. Mireille B Toledano: Supervision, Writing – review & editing. James Nimo: Data curation, Writing – review & editing. Josephine Bedford Moses: Data curation, Writing – review & editing. Solomon Baah: Data curation, Writing – review & editing. Allison Hughes: Data curation, Resources, Writing – review & editing. Alicia Cavanaugh: Data curation, Formal analysis, Writing – review & editing. Samuel Agyei-Mensah: Data curation, Resources, Writing – review & editing. George Owusu: Resources, Writing – review & editing. Brian Robinson: Data curation, Writing – review & editing. Jill Baumgartner: Methodology, Writing – review & editing. James Bennett: Conceptualization, Formal analysis, Methodology, Supervision, Software, Writing – review & editing. Raphael Arku: Conceptualization, Project administration, Supervision, Writing – review & editing.

## Funding sources

This work was supported by the Pathways to Equitable Healthy Cities grant from the Wellcome Trust [209376/Z/17/Z]. For the purpose of open access, the authors have applied a CC BY public copyright license to any Author Accepted Manuscript version arising from this submission. SC was supported by a Canadian Institutes for Health Research (CIHR) PhD scholarship as well as an Imperial College Presidents PhD scholarship. Infrastructure support for the Department of Epidemiology and Biostatistics at Imperial College was provided by the NIHR Imperial Biomedical Research Centre (BRC).

## Declaration of competing interest

The authors declare that they have no known competing financial interests or personal relationships that could have appeared to influence the work reported in this paper.
