# Supplementary material for: Spatial modelling and inequalities of environmental noise in Accra, Ghana
Source: Environ Res. 2022 Nov;214:113932. doi: 10.1016/j.envres.2022.113932 (PMC9441709; doi:10.1016/j.envres.2022.113932)
Supplement: Multimedia component 1 [file mmc1.docx]

**Supplementary Information**

**Spatial modelling and inequalities of environmental noise in Accra, Ghana**

Sierra N Clark ^a,b^, Abosede S Alli ^c^, Majid Ezzati ^a,b,d,e^, Michael Brauer ^f^, Mireille B Toledano ^a,b,g^, James Nimo ^h^, Josephine Bedford Moses ^h^, Solomon Baah ^h^, Allison Hughes ^h^, Alicia Cavanaugh ^i^, Samuel Agyei-Mensah ^j^, George Owusu ^k^, Brian Robinson ^i^, Jill Baumgartner ^l,m^, James Bennett ^a,b^* & Raphael Arku ^c^*

^a^ Department of Epidemiology and Biostatistics, School of Public Health, Imperial College London, London, UK

^b^ MRC Centre for Environment and Health, School of Public Health, Imperial College London, London, UK

^c^ Department of Environmental Health Sciences, School of Public Health and Health Sciences, University of Massachusetts, Amherst, USA

^d^ Regional Institute for Population Studies, University of Ghana, Accra, Ghana

^e^ Abdul Latif Jameel Institute for Disease and Emergency Analytics, Imperial College London, London, UK

^f^ School of Population and Public Health, The University of British Columbia, Vancouver, Canada

^g^ Mohn Centre for Children’s Health and Wellbeing, School of Public Health, Imperial College London, London, UK

^h^ Department of Physics, University of Ghana, Accra, Ghana

^i^ Department of Geography, McGill University, Montreal, Canada

^j^ Department of Geography and Resource Development, University of Ghana, Accra, Ghana

^k^ Institute of Statistical, Social & Economic Research, University of Ghana, Accra, Ghana

^l^ Institute for Health and Social Policy, McGill University, Montreal, Canada

^m^ Department of Epidemiology, Biostatistics, and Occupational Health, McGill University, Montreal, Canada

*****Joint senior authorship

**Pages:** 14

**Tables:** 6

**Figures:** 3

**Equations:** 1

**
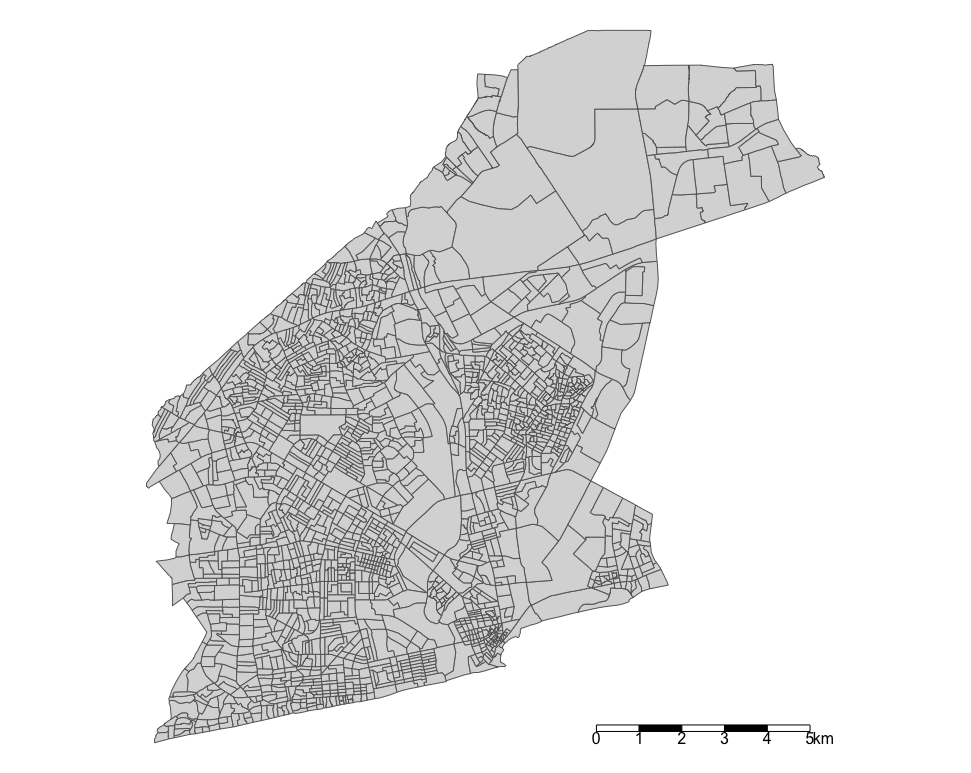
**

**Figure S1. Map of census 2010 enumeration area boundaries in Accra Metropolis.**

**Equation S1. Intermittency ratio calculation**

IR equation re-created from (Wunderli et al., 2016)

Equation A

$$L_{eq,T,Tot}=10*log10(\frac{1}{T}\int_{0}^{T} {10}^{0.1L\left( t \right)}dt)[dB]$$

Equation B

$$K=L_{eq,T,Tot}+C[dB]$$

Equation C

$$L_{eq,T,Events}=10*Log10(\frac{1}{T}\int_{0}^{T} H\left( L\left( t \right)-K \right){10}^{0.1L\left( t \right)}dt)[dB]$$

Equation D

$$IR=\frac{{10}^{0.1L_{eq,T,Events}}}{{10}^{0.1L_{eq,T,Tot}}}*100$$

*IR :* Intermittency ratio

*L_eq,T,Tot_* : Overall sound pressure level for time period *T*

*L_eq,T,Events_* : Event based sound pressure level for time period *T*

*L(t)* : Continuous sound pressure level at the receiver position

*K* : Defined relative to the long-term average of the *L_eq,T,Tot_* and an offset *C*

*C* : Fixed offset above *L_eq,T,Tot_*

*H* : Heaviside step function

The free parameter C was set to +3 dBA above the L_eq,T,tot_ for our study based on earlier work that had been done (Wunderli et al., 2016) which showed that C=3 resulted in a fairly uniform spread of IR across a range of exposure situations. We also calculated and reported IRs from our Accra data with C = 4 and C = 5 in a previous publication (Clark et al., 2021). IR’s that were calculated with C=3, C=4, and C=5 were correlated (C(3) and C(4): r=0.99; C(3) and C(5): r=0.95) across measurement sites and days, though the median IRs were lower when C=4 and C=5, as expected.

**Table S1. Noise level LUR model fit with splines (knots=3)**.**

|  | R^2^ * | Median absolute error * | Mean error * |
| --- | --- | --- | --- |
| LAeq_1hr_ for all day-time hours (dBA) | 0.84 | 1.4 dBA | <0.001 dBA |
| LAeq_1hr_ for all night-time hours (dBA) | 0.84 | 1.7 dBA | <0.001 dBA |

*Model fit/error estimated without cross validation.

**Random effects and predictor variable sets are the same as the main models, however non-linear spline functions were applied to predictor variable associations.

Allowing for non-linear associations of the predictor variables in the final (main) models of LAEq_1hr_ with splines did not improve model R^2^ or error metrics (MAE, ME). Though the marginal fixed effect portion of the R^2^ did improve minimally by 0.03.

**Table S2. Random Forest noise level LUR model fit*.**

|  | No cross validation | | CV_10%sites_ cross-validation | |
| --- | --- | --- | --- | --- |
|  | Median absolute error | Mean error | Median absolute error | Mean error |
| LAeq_1hr_ for all day-time hours (dBA) | 1.4 | <0.01 | 3.7 | 0.10 |
| LAeq_1hr_ for all night-time hours (dBA) | 1.7 | <0.01 | 4.0 | 0.11 |

*Predictor variable sets were the same as the main models. The variable for ‘hour’ was included as a fixed effect in the LAeq_1hr_ random forest models.

**Table S3. Mean associations of LAeq_1hr_ with temperature, relative humidity, wind speed and rainfall in bivariate and multivariable mixed-effect linear regression LUR models*.**

|  | Unadjusted mean associations  (bivariate models) | | Adjusted mean associations (multivariable models with weather predictor variables) | | Adjusted mean associations (multivariable models with spatial and weather predictor variables) | |
| --- | --- | --- | --- | --- | --- | --- |
|  | **Day-time hours LAeq_1hr_ model** | **Night-time hours LAeq_1hr_ model** | **Day-time hours LAeq_1hr_ model** | **Night-time hours LAeq_1hr_ model** | **Day-time hours LAeq_1hr_ model**** | **Night-time hours LAeq_1hr_ model***** |
|  | Slope coefficient  [95% CI] | Slope coefficient  [95% CI] | Slope coefficient  [95% CI] | Slope coefficient  [95% CI] | Slope coefficient  [95% CI] | Slope coefficient  [95% CI] |
| Hourly temperature *Standardized (degrees Celsius)* | -0.44 [-0.48, -0.40] | -0.12 [-0.17, -0.07] | -0.15 [-0.21, -0.10] | -0.01 [-0.06, 0.04] | -0.15 [-0.20, -0.09] | -0.01 [-0.06, 0.04] |
| Hourly relative humidity  *Standardized (%)* | 0.36 [0.33, 0.40] | 0.12 [0.07, 0.16] | 0.18 [0.13, 0.23] | 0.06 [0.02, 0.11] | 0.18 [0.13, 0.23] | 0.06 [0.02, 0.11] |
| Hourly wind speed *Standardized (m/s increase)* | 0.43 [0.39, 0.46] | 0.26 [0.22, 0.31] | 0.36 [0.33, 0.39] | 0.24 [0.19, 0.29] | 0.36 [0.33, 0.39] | 0.24 [0.19, 0.28] |
| Daily rainfall *Standardized (mm)* | 0.12 [0.09, 0.15] | 0.09 [0.04, 0.13] | 0.06 [0.04, 0.09] | 0.05 [0.01, 0.10] | 0.06 [0.04, 0.09] | 0.05 [0.01, 0.09] |

95% CI: 95% confidence interval; Standardized: Weather variables standardized by subtracting each value by the data mean and dividing by the data standard deviation.

* All models have random efffects for *site* and *hour of the day*.

** Spatial predictor variables: NDVI, length of major roads, number of restaurants, length of secondary/tertiary roads, formal residential land use area, population density, height of monitor off the ground.

***Spatial predictor variables: Length of major roads, NDVI, number of restaurants, length of secondary/tertiary roads, formal residential land use area, height of monitors off the ground.

Data on hourly temperature, wind speed, and relative humidity that we collected at the 6 fixed measurement sites were averaged across the sites to obtain a single date specific hourly value for the study area. We also used daily rainfall data from the Ghana Meteorological Agency that represented the entire area.

We first estimated the unadjusted mean associations between LAeq_1hr_ and hourly average temperature, relative humidity, and wind speed and daily average rainfall, matched on date and time of measurement. We then added the weather variables into the main spatial models and estimated the adjusted mean associations with LAeq_1hr_. We also investigated whether the predictive accuracy of the main spatial models improved with the inclusion of the weather variables.

The ways in which atmospheric conditions can affect measured sound levels are complex. Previous research shows that atmospheric propagation of sound waves can be impacted by wind speed, temperature, and humidity (Kang, 2017). As well, sound from other sources can be amplified under certain atmospheric conditions, such as how motor vehicle sounds from the contact of tires with the road-material is amplified in wet conditions (Kang, 2017). Finally, atmospheric conditions such as high winds or rain can be sources of sound energy themselves, and they can influence human behaviours/activities that result in sound generation (Böcker et al., 2013).

Wind speed, rainfall, and humidity were positively associated with LAeq_1hr_, while temperature had the inverse relationship. In all cases, the magnitude of the associations between LAeq_1hr_ and weather variables in the day and night-time were fairly minimal, which is supported by the fact that atmospheric attenuations of short-range sound propagation are minimal (Kang, 2017). Among the various weather parameters considered, wind speed had the largest magnitude of effect. This is expected as for short-range sound propagation, the effects of wind structure (i.e., wind speed) are more important than other atmospheric conditions (Kang, 2017). Higher winds could be assisting in the propagation of sound from sources towards the sound level meters (receivers). The observed positive association between relative humidity and LAeq_1hr_ has been found in a previous noise LUR study in Taichung (Taiwan), but only at a higher frequency level (8000 Hz for LAeq_24hr_) (Chang et al., 2019). The relationship between sound wave attenuation and relative humidity is complex as the relationship changes with temperature and sound frequency. Though at higher relative humidity levels (i.e., >70 % as is the case in Accra), increasing humidity has been shown to lead to a decrease in atmospheric attenuation (Mahdi and Al-jumaily, 2018). This relationship has been found at higher temperature levels, 25-35 degrees Celsius (Mahdi and Al-jumaily, 2018), as is the case in Accra.

Inclusion of time-resolved weather data did not improve the predictive accuracy of the main models. The median absolute prediction error (MAE) of the main spatial day and night-time LAeq_1hr_ models only changed by 0.01 dBA when weather predictor variables were included. The coefficients of the spatial variables remained relatively unchanged with the inclusion of the weather variables.

**Table S4. Mean associations between noise levels and spatial predictor variables in bivariate LUR models.**

| Variables | Buffer radii (m) | LAeq_1hr_ for all day-time hours (dBA)  Coefficient  [95% CI] | Buffer radii (m) | LAeq_1hr_ for all night-time hours (dBA)  Coefficient  [95% CI] |
| --- | --- | --- | --- | --- |
| Transportation |  |  |  |  |
| Distance to nearest major road *(sqrt(meters))* | - | -0.13 [-0.17, -0.09] | - | -0.11 [-0.16, -0.07] |
| Distance to nearest secondary/ tertiary road *(sqrt(meters))* | - | -0.25 [-0.32, -0.17] | - | -0.25 [-0.33, -0.17] |
| Distance to airport  *(sqrt(meters))* | - | -0.08 [-0.10, -0.05] | - | -0.07 [-0.09, -0.04] |
| Near the airport (inside a 1km buffer) *(Yes, ref No)* | - | 0.11 [-6.92, 7.14] | - | 3.44 [-3.64, 10.52] |
| Total length of all roads *(standardized meters)* | 100 | 1.95 [0.57, 3.36] | 100 | 1.76 [0.34, 3.17] |
| Total length of major roads *(standardized meters)* | 100 | 4.50 [2.82, 6.18] | 100 | 5.19 [3.54, 6.84] |
| Total length of secondary and tertiary roads *(standardized meters)* | 200 | 3.90 [2.72, 5.09] | 200 | 4.23 [3.06, 5.40] |
| Total length of minor roads *(standardized meters)* | 100 | -1.72 [-2.66, -0.86] | 100 | -2.26 [-3.14, -1.38] |
| Presence of bus station/terminal *(Yes, ref No)* | 200 | 7.59 [-0.89, 16.09] | 200 | 7.59 [-0.98, 16.17] |
| Land use and vegetation |  |  |  |  |
| Total informal high-density residential area *(standardized meters^2^)* | 200 | 2.63 [1.42, 3.87] | 200 | 2.03 [0.77, 3.28] |
| Total formal (medium/low-density) density residential area *(standardized meters^2^)* | 200 | -0.54 [-1.55, 0.47] | 200 | -0.95 [-1.96, 0.06] |
| Total CBI area *(standardized meters^2^)* | 50 | 2.18 [1.34, 3.29] | 50 | 2.04 [0.96, 3.10] |
| Total ‘other’ area *(standardized meters^2^)* | 500 | -2.29 [-3.34, -1.64] | 500 | -1.76 [-2.66, -0.85] |
| Average NDVI *(standardized value [0-1])* | 50 | -3.82 [-4.51, -3.13] | 50 | -3.01 [-3.80, -2.21] |
| Length of waterways *(standardized meters)* | 200 | 0.52 [0.02, 1.04] | 100 | 0.44 [-0.04, 0.93] |
| Elevation above sea-level *(meters)* | - | -0.07 [-0.10, -0.04] | - | -0.05 [-0.08, -0.01] |
| Population and building density |  |  |  |  |
| Average population density *(standardized pop/km^2^)* | 500 | 3.90 [2.62, 5.17] | 500 | 3.05 [1.70, 4.40] |
| Total number of buildings *(standardized count)* | 500 | 3.12 [2.15, 4.10] | 100 | 2.40 [1.12, 3.67] |
| Locations of human activity |  |  |  |  |
| Presence of mosque(s) *(Yes, ref No)* | 500 | 4.98 [2.72, 7.19] | 500 | 3.67 [1.36, 5.96] |
| Number of restaurants *(Count)* | 100 | 3.07 [1.77, 4.37] | 100 | 3.21 [1.90, 4.51] |
| Number of churches *(Count)* | 100 | 1.00 [-0.33, 2.34] | 100 | -0.03 [-1.4, 1.32] |
| Presence of a hospital  *(Yes, No)* | 500 | 2.59 [-1.77, 6.95] | 500 | 3.02 [-1.37, 7.42] |
| Presence of a school  *(Yes, ref No)* | 500 | 6.08 [3.66, 8.49] | - | - |
| Presence of shopping mall(s) *(Yes, ref No)* | 200 | 5.71 [3.28, 8.15] | - | - |
| Prescence of a bar or nightclub *(Count)* | - | - | 500 | 0.42 [0.13, 0.70] |

CBI: Commercial, business, industrial; CI: Confidence interval. LAeq_1hr_ models had random intercepts for *hour* and *site*.


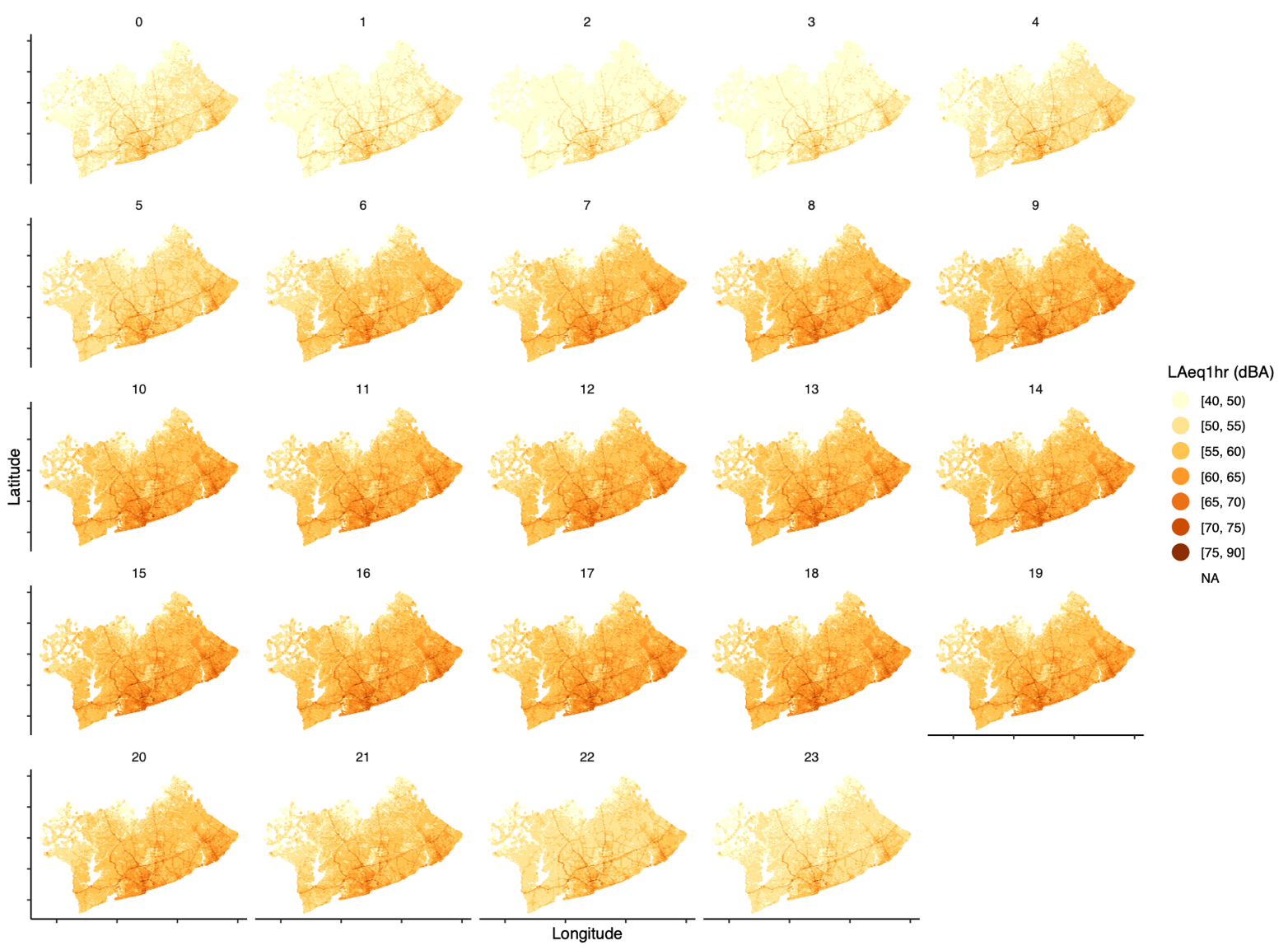


Figure S2. Predicted annual average LAeq_1hr_ for each hour of the day. Predictions were made onto a 50m x 50m spatial resolution grid for a fixed height of 4 meters off the ground. White ‘NA’ areas are excluded from prediction as they are considered out of sample (e.g., water bodies, forests, grasslands). Numbers above maps refer to the hour of the day based on the 24-hour clock (e.g., 2: 2am).

**Table S5. Number and percentage of the population living in enumeration areas (EA) with varying levels of average L_den_ and L_night_ in Accra Metropolis.** Population distributions within EAs are based on the most recent (2010) 100% sample of the national census.

| Noise band | L_den_ | | L_night_ | |
| --- | --- | --- | --- | --- |
|  | **Percentage of the population** | **Number of people** | **Percentage of the population** | **Number of people** |
| 45-49 dBA | - | - | Less than 1% | 9,630 |
| 50-54 dBA | - | - | 32% | 536,179 |
| 55-59 dBA | 2% | 35,076 | 54% | 888,181 |
| 60-64 dBA | 31% | 515,873 | 12% | 201,622 |
| 65-69 dBA | 53% | 876,098 | 1% | 15,432 |
| 70-74 dBA | 13% | 216,458 | Less than 1% | 2,328 |
| 75+ dBA | Less than 1% | 9,867 | - | - |

**
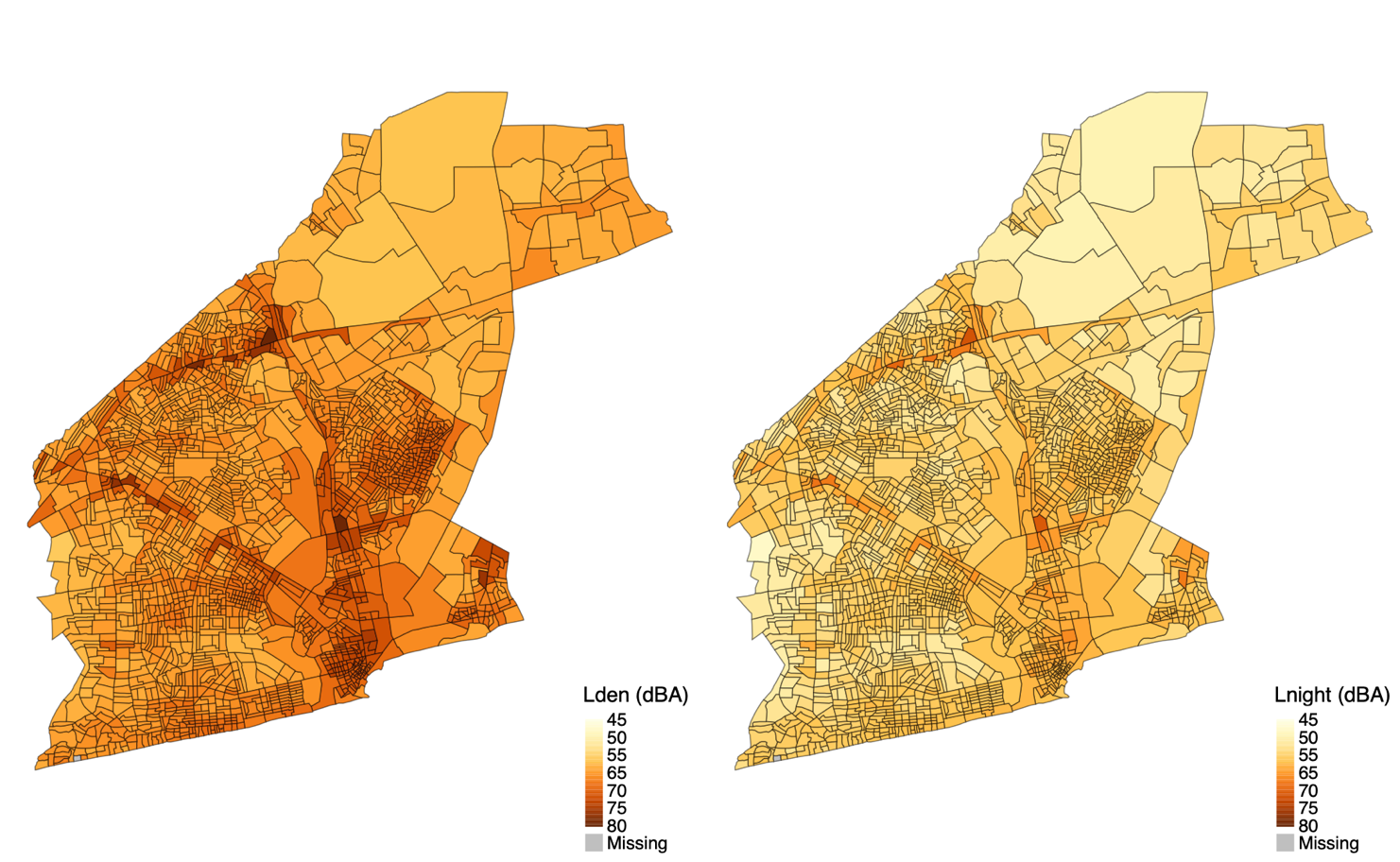
**

**Figure S3. Average predicted L_den_ and L_night_ within enumeration areas (EAs) in Accra Metropolis.**

**Table S6.** **Intermittency Ratio (IR) regression models*.**

| Predictor variable  (buffer radii size) | Units | IR_day_ model | IR_night_ model |
| --- | --- | --- | --- |
|  |  | **Coefficients**  [95% Confidence Interval] | **Coefficients**  [95% Confidence Interval] |
| Total length of major roads (100m) | Standardized meters | -4.62 [-9.90, 0.67] | -5.55 [-10.49, -0.61] |
| Total length of secondary/tertiary roads (200m) | Standardized meters |  | -2.68 [-6.47, 1.10] |
| Total length of minor roads (200m) | Standardized meters | 6.55 [3.73, 9.37] |  |
| Normalized difference vegetation index (100m) | Standardized value | 9.67 [5.61, 13.75] |  |
| *Interaction terms* |  |  |  |
| L_day_: Commercial, business, industrial area | dBA | 1.78 [1.62, 1.94] |  |
| L_day_: High-density residential area | dBA | 2.22 [2.07, 2.37] |  |
| L_day_: Low-density residential area | dBA | 2.28 [2.14, 2.42] |  |
| L_day_: Peri-urban background site | dBA | 2.36 [2.22, 2.49] |  |
| L_night:_ Commercial, business, industrial area | dBA |  | 1.74 [1.58, 1.90] |
| L_night_: High-density residential area | dBA |  | 1.94 [1.81, 2.08] |
| L_night_: Low-density residential area | dBA |  | 2.11 [1.95, 2.27] |
| L_night_: Peri-urban background site | dBA |  | 2.16 [2.01, 2.33] |
|  |  |  |  |
|  |  | **R^2^ (fixed-effect component)** | **R^2^ (fixed-effect component)** |
|  |  | 0.54 ** | 0.32 ** |
|  |  | **R^2^ (fixed and random components)**  0.84 ** | **R^2^ (fixed and random components)**  0.63 ** |
|  |  | **Range of Variance Inflation factors** | **Range of Variance Inflation factors** |
|  |  | 1.23 – 3.05 | 1.13 – 1.62 |

*All models had random effects for *site*. Continuous predictor variables were standardized by subtracting by the data mean and dividing by the data standard deviation. A 1-point change in a standardized variable corresponds to a 1 standard deviation increase on the original scale.

**Model fit (R^2^) estimated without cross validation.

**References**

Böcker, L., Dijst, M., Prillwitz, J., 2013. Impact of Everyday Weather on Individual Daily Travel Behaviours in Perspective: A Literature Review. Transp. Rev. 33, 71–91. https://doi.org/10.1080/01441647.2012.747114

Cavanaugh, A., 2021. Poverty & Inequality Mapping in Ghana, in: Pathways to Equitable Healthy Cities Consortium Meeting.

Chang, T.Y., Liang, C.H., Wu, C.F., Chang, L. Te, 2019. Application of land-use regression models to estimate sound pressure levels and frequency components of road traffic noise in Taichung, Taiwan. Environ. Int. 131, 104959. https://doi.org/10.1016/j.envint.2019.104959

Clark, S.N., Alli, A.S., Nathvani, R., Huges, A., Ezzati, M., Brauer, M., Toledano, M.B., Baumgartner, J., Bennett, J.E., Nimo, J., Moses, J.B., Baah, S., Agyei-Mensah, S., Owusu, G., Croft, B., Arku, R.E., 2021. Space-time characterization of community noise and sound sources in Accra, Ghana. Sci. Rep. 1–12. https://doi.org/https://doi.org/10.1038/s41598-021-90454-6

Kang, J., 2017. Urban Sound Environment, 1st ed. Routledge.

Mahdi, Z.S., Al-jumaily, K.J., 2018. Atmospheric effects on sound waves propogation. Iraqi J. Sci. 53, 689–692.

Truax, B. (Ed.), 1999. Sound Propagation, in: Handbook for Acoustic Ecology.

Wunderli, J.M., Pieren, R., Habermacher, M., Vienneau, D., Cajochen, C., Probst-Hensch, N., Röösli, M., Brink, M., 2016. Intermittency ratio: A metric reflecting short-term temporal variations of transportation noise exposure. J. Expo. Sci. Environ. Epidemiol. 26, 575–585. https://doi.org/10.1038/jes.2015.56
